# Supplementary material for: Collaborative intelligence in AI: Evaluating the performance of a council of AIs on the USMLE
Source: PLOS Digit Health. 2025 Oct 9;4(10):e0000787. doi: 10.1371/journal.pdig.0000787 (PMC12510544; doi:10.1371/journal.pdig.0000787)
Supplement: S2 File — (ZIP) [file pdig.0000787.s002.zip › S2 Data Raw data files containing unprocessed question inputs and LLM outputs including transcripts of deliberations/STEP3_Question-31-1_Type-MC-NJ.docx]

**Facilitator** **[Round 1]:** A 16-month-old girl is brought to the emergency department by emergency medical technicians because of a generalized tonic-clonic seizure that began 25 minutes ago and has continued despite administration of 0.5 mg/kg rectal diazepam 10 minutes ago. The patient was at home at the time of the seizure. In the emergency department, she is given an additional 0.5 mg/kg dose of diazepam intravenously and the convulsion terminates. Within 2 minutes, her oxygen saturation drops to 75% and she appears cyanotic. Respirations are 10/min and shallow. She receives bag-valve-mask ventilation, followed by intubation and mechanical ventilation. Medical history is unremarkable. She takes no medications. Temperature is 39.1°C (102.5°F). Which of the following is the most likely underlying cause of the patient's respiratory insufficiency? (A) Airway occlusion (B) Encephalitis (C) Medication-induced suppression of central respiratory drive (D) Meningitis (E) Ongoing nonconvulsive seizure activity

**AI_E:** RESTATING THE QUESTION: A 16-month-old girl has experienced a prolonged seizure, was treated with diazepam, but then became hypoxic and required assisted ventilation. She has no significant medical history and takes no daily medications. She also has a fever. We are being asked to identify the most probable cause of her respiratory issues from a list of possible causes, including blocked airways, encephalitis, drug-caused central respiratory drive suppression, meningitis, and ongoing nonconvulsive seizure activity. PLANNING: Now let's identify the assumptions. The assumption is that the patient's respiratory insufficiency is due to one of the listed causes. However, it is also important to consider that the underlying cause can be a combination of these factors. CLINICAL REASONING: The girl's oxygen saturation dropping and the appearance of cyanosis would support respiratory depression. Her shallow breathing would also imply that her brain isn't effectively signaling her to breathe deeply, which may indicate a central respiratory drive issue. Furthermore, the timing, coming after the diazepam administration, suggests that this drug, known for the side effect of respiratory suppression, could have induced this. Encephalitis and meningitis typically present with other neurological signs, and we don't have strong evidence to support these diagnoses here. Airway occlusion might cause sudden respiratory distress, but it is less likely in this context and we have no mentioned signs of a great obstruction, such as stridor. Lastly, continuing nonconvulsive seizure activity might possibly cause her condition, but it's less likely considering the convulsion stopped with diazepam administration. CONCLUDING: Referring to the clinical reasoning process and the given information, the fact that the infant's respiratory depression took place after repeated doses of diazepam (a benzodiazepine known to suppress central respiratory drive) suggests that her respiratory insufficiency may have been induced by the medication. RESPONSE: The patient's respiratory insufficiency is most likely induced by the suppression of central respiratory drive from the diazepam (Choice C: Medication-induced suppression of central respiratory drive).

**AI_C:** RESTATING THE QUESTION: A 16-month-old girl who was previously healthy and currently taking no medications, comes to the emergency department following a generalized tonic-clonic seizure that began 25 minutes ago. Despite original treatment with rectal diazepam, the seizure persisted requiring further intervention with an additional intravenous dose. After terminating the seizure, the patient's oxygen saturation drops significantly, and she exhibits signs of respiratory distress, becoming cyanotic with shallow breathing. After securing the airway with mechanical ventilation, it's noticed that her temperature is elevated. In this situation, we're asked to identify what is the most likely cause of her respiratory insufficiency, given the options of airway occlusion, encephalitis, medication-induced suppression of central respiratory drive, meningitis, or ongoing nonconvulsive seizure activity. PLANNING: Let's review each assumption. (A) Airway occlusion may cause cyanosis and respiratory issues but she is already intubated hence it is unlikely. (B) Encephalitis is inflammation of the brain, often caused by a viral infection. An important symptom is high fever which present in this case. (C) Medication-induced suppression of central respiratory drive is possible with benzodiazepines like diazepam but two doses are unlikely to cause severe respiratory suppression in a healthy child without any other medications; (D) Meningitis - the symptoms often include fever and seizures but wouldn't cause such rapid respiratory collapse; (E) Ongoing nonconvulsive seizure activity would not typically cause respiratory collapse. CLINICAL REASONING: Among these assumptions, we can therefore clinically prioritize the likelihoods as follows: (C) Medication-induced suppression of central respiratory drive needs to be examined more carefully. Diazepam, a benzodiazepine, enhances the effect of the neurotransmitter gamma-aminobutyric acid (GABA) at the GABA_A receptor, resulting in sedative, hypnotic (sleep-inducing), anxiolytic, anticonvulsant, and muscle relaxant properties. High doses, especially when injected rapidly, can cause significant central nervous system depression, including respiratory depression. CONCLUDING: While encephalitis and meningitis may cause seizures and fever, and airway occlusion could explain a decrease in oxygen saturation, it is unlikely for these conditions to manifest as rapidly progressive respiratory failure in the timeline described. However, the administration of multiple doses of a potent central nervous system depressant in a short span is a plausible explanation for the rapid onset of respiratory depression and consequential oxygen desaturation. RESPONSE: Given the information presented, it seems that the most likely cause of the patient's respiratory insufficiency is (C) Medication-induced suppression of central respiratory drive.

**AI_A:** RESTATING THE QUESTION: In order to determine the likely underlying cause for the noted respiratory insufficiency in this 16-month-old girl, it's important to understand the sequence of events. The patient presents with a history of a generalized tonic-clonic seizure that lasted for 25 minutes and was not initially responsive to rectal diazepam. She was given another dose of diazepam intravenously in the emergency department where the convulsion was finally terminated. Shortly after, she displayed signs of respiratory distress like cyanosis and shallow breathing. The suggestions for the cause of the respiratory difficulty are airway occlusion, encephalitis, medication-induced suppression of respiratory drive, meningitis, and ongoing nonconvulsive seizure activity. PLANNING: Given the uninterrupted seizure activity and the subsequent presentation, several assumptions can be made. Firstly, pathologies such as encephalitis and meningitis could potentially lead to prolonged seizure activity, however, they typically present with additional neurological symptoms not reported here. Medication-induced suppression of the respiratory drive could also be an assumption considering the administration of diazepam, a known respiratory depressant. Similarly, nonconvulsive seizure activity could be continuing despite apparent cessation of convulsions. Lastly, although an airway occlusion can cause hypoxia and cyanosis, in this context, it is less probable without a known or observed event of aspiration or airway obstruction. Notably, the patient also received bag-valve-mask ventilation and intubation, which would likely have resolved an airway occlusion. Therefore, given the absence of other neurological signs and the salient feature of respiratory depression post-diazepam administration, the mostly likely assumption is that the patient is experiencing a medication-induced suppression of the central respiratory drive. CLINICAL REASONING: Diazepam, just like other benzodiazepines, exert their effect by enhancing the GABA, the major inhibitory neurotransmitter in the central nervous system. GABA activation decreases neuronal excitability, hence the use of benzodiazepines in controlling seizures. However, it also is responsible for controlling certain autonomous functions, including respiratory drive. An overdose or a high dose of benzodiazepine can potentially suppress the central respiratory drive leading to hypoventilation, hypoxia and eventual cyanosis as we are seeing in this patient. In addition, the symptoms presented immediately following repeated diazepam administration, which strengthens this notion. The presence of fever would fit with a post-ictal state, since a seizure could lead to increased body temperature due to muscle contractions and increased metabolic activity. CONCLUDING: The patient's sudden respiratory insufficiency and cyanosis occurred immediately after the administration of a second dose of diazepam, indicating that a medication-induced suppression of central respiratory drive is the most likely cause. RESPONSE: The most likely underlying cause for the patient's respiratory insufficiency is (C) Medication-induced suppression of central respiratory drive.

**AI_D:** RESTATING THE QUESTION: This situation involves a 16-month-old girl who was taken to the emergency department due to a prolonged generalized tonic-clonic seizure. Despite the administration of rectal diazepam, the seizure persisted and after an additional dose of diazepam was given intravenously, the seizure stopped. However, her oxygen saturation dropped sharply, her respirations became shallow and she turned blue (cyanotic). After intubation and ventilation, the question is to identify the most probable cause of her respiratory insufficiency. Possible reasons presented are airway occlusion, encephalitis, medication-induced suppression of central respiratory drive, meningitis, or ongoing nonconvulsive seizure activity. PLANNING: The assumptions to generate here are linked to each option given. We first have to exclude airway occlusion because after a thorough airway assessment, we've seen that she's been successfully intubated and ventilated. Then, we have to examine the probability of encephalitis and meningitis – these conditions usually present with a seizure, but their significant signs like altered consciousness and neck stiffness are not mentioned here. In addition, these are not commonly visualized causing acute respiratory insufficiency. Also, the ongoing nonconvulsive seizure activity doesn't usually cause severe hypoxia. Lastly, we need to focus on medication-induced suppression of central respiratory drive. Diazepam, as a benzodiazepine, is known for its CNS depressive actions, including suppression of the respiratory drive. This patient received two doses of diazepam successively, increasing the chances it may be causing her respiratory difficulties. CLINICAL REASONING: Despite the causes in the options, it's critical to connect our clinical information with each assumption. Airway obstruction could be ruled out due to successful intubation and ventilation. The picture doesn't fit encephalitis and meningitis significantly due to lack of typical findings. Nonconvulsive seizure activity isn't strongly linked to severe respiratory compromise. The girl has received significant amounts of diazepam, given as a benzodiazepine medication, which is known for its ability to suppress the central respiratory drive. This drugs' effect would be directly proportional to the dosage given and the more diazepam given, the higher her chances of respiratory difficulties. CONCLUDING: Given the systematic exclusion of other differential diagnoses and the strong correlation between the administration of diazepam and the clinical picture of respiratory insufficiency, it leads to the conclusion that the main contributing factor is most likely diazepam inducing suppression of central respiratory drive. RESPONSE: Therefore, the most likely underlying cause of the patient's respiratory insufficiency is (C) Medication-induced suppression of central respiratory drive.

**AI_B:** The scenario provided pertains to a 16-month-old girl who suffered from a generalized tonic-clonic seizure. Despite receiving rectal diazepam, the seizure persisted. An additional dose of diazepam was administered intravenously in the emergency department that ceased convulsion. Not long later, her oxygen levels drop significantly, and she was cyanotic. The breathing pattern became shallow and slower. Following bag-valve-mask ventilation, she was intubated for mechanical ventilation. This child had an unnotable medical history and was not on any medication prior to this episode. Her body temperature was elevated. The problem at hand is determining the most probable reason for this child's respiratory insufficiency. Tonic-clonic seizures, also previously known as grand mal seizures, are characterized by muscle stiffness (tonus) and jerking movements (clonus). Diazepam is a benzodiazepine used to temporarily control seizure episodes. Via enhancing inhibitory GABA neurotransmission, it helps reduce the over-excitability in the brain that leads to seizures. Cyanosis indicates an insufficient amount of oxygen in the blood. We should consider multiple possibilities regarding the cause of the respiratory insufficiency evident from the lower oxygen saturation levels, cyanosis, and shallow respiration. These could be due to airway blockage, encephalitis, medication-induced suppression of central respiratory drive, meningitis, or continuing nonconvulsive seizure activity. The noted seizure activity could suggest encephalitis or meningitis. Encephalitis is the inflammation of the brain often a result of viral infection causing fever, seizures, among other symptoms. Similarly, meningitis is the inflammation of the meninges, the protective membranes of the brain and spinal cord, usually due to infectious agents. It can present with elevated temperature, headache, and seizures. Nonconvulsive seizure activity, although terminated visibly, might still be occurring electrically inside the brain. Diazepam and similar drugs, while useful for stopping seizures, can have the potential side effect of suppressing the brain's independent control over respiration, leading to a slower breathing rate and lesser oxygen uptake. Bearing these alternate options in mind, we delve deeper into each assumption hypothesizing the most clinically relevant cause. For airway occlusion, considering the successful intubation and initiation of mechanical ventilation, the possibility of an obstructed airway becomes less likely. Let's evaluate the clinical reasoning behind deducting the most likely cause. The timing of events suggests a link between the diazepam administration and the child's respiratory insufficiency, with the shallow respirations and cyanosis appearing after the drug was given. This lends credence to the possibility of a medication-induced suppression of the central respiratory drive. Diazepam’s therapeutic effects may simultaneously depress the patient's central respiratory drive, slowing down their breathing rate. This makes it the most plausible cause, relative to the other explanations. We also consider the fever. In meningitis and encephalitis, fever is commonly present and often accompanies seizures due to the illness's inflammatory nature. However, in this case, the seizures were successfully terminated with diazepam, making ongoing nonconvulsive seizure activity a less likely cause of the respiratory insufficiency. To conclude, my analysis based on a step-by-step clinical thought process. The most likely cause of the patient's respiratory insufficiency is the medication-induced suppression of the central respiratory drive. The correlation between the administration of diazepam and the subsequent fall in the patient’s oxygen saturation levels, together with her cyanosis and reduced respiration rate, highly suggest this. Hence, (C) Medication-induced suppression of the central respiratory drive, is the most probable underlying cause of this 16-month-old girl's respiratory insufficiency.

**Facilitator** The consensus statement synthesizes the responses of AI_A, AI_B, AI_C, AI_D, and AI_E as follows: The case involves a 16-month-old girl who experienced a prolonged generalized tonic-clonic seizure. Despite the administration of rectal diazepam, the seizure persisted, and an additional dose of diazepam was given intravenously, which terminated the seizure. However, her oxygen saturation dropped significantly, her respirations became shallow, and she turned cyanotic. After intubation and ventilation, the task is to identify the most probable cause of her respiratory insufficiency. The possible causes presented are airway occlusion, encephalitis, medication-induced suppression of central respiratory drive, meningitis, or ongoing nonconvulsive seizure activity. Airway occlusion is unlikely due to successful intubation and ventilation. Encephalitis and meningitis, while they can cause seizures and fever, are not typically associated with rapid respiratory failure in the timeline described. Ongoing nonconvulsive seizure activity is also less likely as it doesn't usually cause severe hypoxia. The most plausible cause is medication-induced suppression of central respiratory drive. Diazepam, a benzodiazepine, is known for its central nervous system depressive actions, including suppression of the respiratory drive. This patient received two doses of diazepam successively, which could have led to her respiratory difficulties. The timing of the respiratory depression, coming after the diazepam administration, further supports this conclusion. Therefore, the consensus is that the most likely underlying cause of the patient's respiratory insufficiency is medication-induced suppression of central respiratory drive.
